# Neuroscience of Virtual Reality: From Virtual Exposure to Embodied Medicine

**DOI:** 10.1089/cyber.2017.29099.gri

**Published:** 2019-01-14

**Authors:** Giuseppe Riva, Brenda K. Wiederhold, Fabrizia Mantovani

**Affiliations:** ^1^ Applied Technology for Neuro-Psychology Lab, IRCCS Istituto Auxologico Italiano, Milan, Italy.; ^2^ Department of Psychology, Università Cattolica del Sacro Cuore, Milan, Italy.; ^3^ Virtual Reality Medical Center, La Jolla, California.; ^4^ Virtual Reality Medical Institute, Brussels, Belgium.; ^5^ Department of Human Sciences for Education, Università degli Studi di Milano-Bicocca, Milan, Italy.

## Abstract

Is virtual reality (VR) already a reality in behavioral health? To answer this question, a meta-review was conducted to assess the meta-analyses and systematic and narrative reviews published in this field in the last twenty-two months. Twenty-five different articles demonstrated the clinical potential of this technology in both the diagnosis and the treatment of mental health disorders: VR compares favorably to existing treatments in anxiety disorders, eating and weight disorders, and pain management, with long-term effects that generalize to the real world. But why is VR so effective? Here, the following answer is suggested: VR shares with the brain the same basic mechanism: embodied simulations. According to neuroscience, to regulate and control the body in the world effectively, the brain creates an embodied simulation of the body in the world used to represent and predict actions, concepts, and emotions. VR works in a similar way: the VR experience tries to predict the sensory consequences of an individual's movements, providing to him/her the same scene he/she will see in the real world. To achieve this, the VR system, like the brain, maintains a model (simulation) of the body and the space around it. If the presence in the body is the outcome of different embodied simulations, concepts are embodied simulations, and VR is an embodied technology, this suggests a new clinical approach discussed in this article: the possibility of altering the experience of the body and facilitating cognitive modeling/change by designing targeted virtual environments able to simulate both the external and the internal world/body.

## Virtual Reality in Behavioral Health: A Meta-Review

**FIG. 1.**
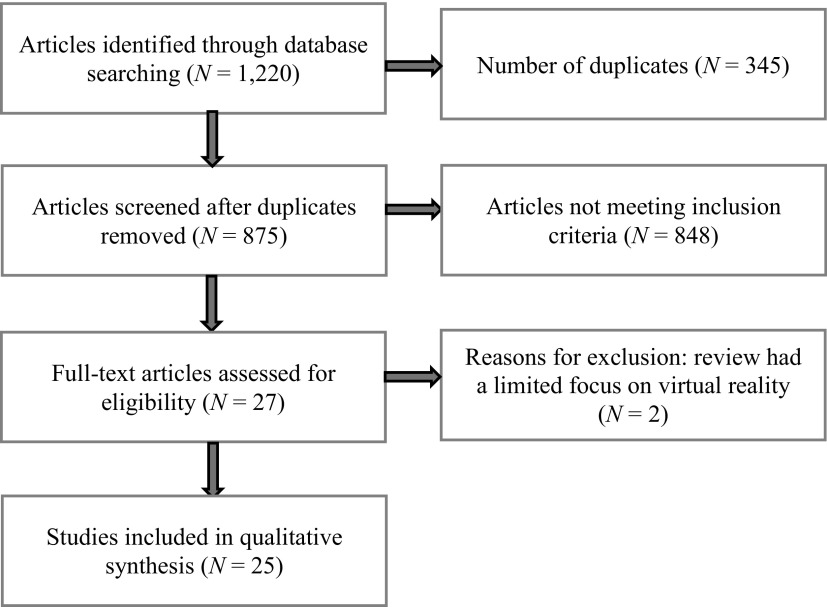
Meta-review methodology. Using the Google Scholar and Scopus databases, a systematic search was conducted to identify reviews (both systematic and narrative) and meta-analyses that reported on the effects of virtual reality (VR) in the assessment and treatment in behavioral health: anxiety disorders, pain management, schizophrenia spectrum disorders, eating and weight disorders, autism spectrum disorders, personality disorders, and substance use disorders. Guidelines for conducting a systematic review discussed by Uman^162^ were followed. The “free-form” question was as follows: “Do virtual environments perform equal-to-or-better-than traditional modalities in behavioral health?” The outcome of interest was reviews and meta-analyses answering this question in any area of behavioral health. The following search terms were used: ((“Virtual Reality” AND (“Review” OR “Meta-analysis” OR “metaanalysis”)) AND (“anxiety” OR “phobia” OR “fear” OR “stress” OR “pain” OR “schizophrenia” OR “psychosis” OR “obesity” OR “eating disorders” OR “bulimia” OR “binge eating” OR “anorexia”, OR “autism” OR “Asperger” OR “substance” OR “drug” OR “nicotine” OR “cocaine” OR “opioids”). The search targeted articles published between November 2, 2016, and August 1, 2018. Inclusion criteria included (a) reviews or meta-analyses, (b) English language journals, and (c) peer-reviewed journals. Exclusion criteria included (a) articles related to the use of VR in surgery or in physical and cognitive rehabilitation; and (b) articles lacking basic information about the selection of the discussed articles. The meta-review flow diagram is shown.

This special issue presented and discussed different virtual reality (VR) applications for behavioral health. But is VR already a reality in behavioral health? To answer this question, a meta-review was conducted to assess the meta-analyses and systematic and narrative reviews (see Fig. 1 for the methodology) published in this field in the last 22 months.

Twenty-five different articles^1–25^ (see Table 1 for the articles' list and a summary of their conclusions) demonstrated the clinical potential of this technology in both the diagnosis and the treatment of mental health disorders. Nine articles^1,2,6,9,14,15,19,18,22^ reviewed the available literature on the effectiveness of VR in psychiatric/mental health treatment.

**Table 1.** Meta-Analyses and Systematic and Narrative Reviews Published in the Last 12 Months Related to the Use of Virtual Reality in the Diagnosis and Treatment of Mental Health Disorders

*Review type*	*Article*	*Included studies*	*Conclusions (from the articles)*
Systematic meta-review	Riva G, Baños RM, Botella C, et al. Transforming experience: the potential of augmented reality and virtual reality for enhancing personal and clinical change. Frontiers in Psychiatry 2016; 7:164.^1^	27 systematic reviews and meta-analyses	“The available data support the use of this technology in the treatment of anxiety disorders, pain management, obesity and eating disorders, and stress-related disorders. But still, there is no clear good quality evidence for or against using VR for the treatment of depression and schizophrenia.”
Systematic review (mental health)	Freeman D, Reeve S, Robinson A, et al. Virtual reality in the assessment, understanding, and treatment of mental health disorders. Psychological Medicine 2017; 47:2393–2400.^2^	285 studies	“VR environments can elicit psychiatric symptoms, manipulation of VR can inform the understanding of disorders, and simpler psychological treatments can be successfully administered in VR. The most established finding is that VR exposure-based treatments can reduce anxiety disorders, but there are numerous research and treatment avenues of promise.”
Reply to the above systematic review (eating and weight disorders)	Riva G. Letter to the editor: virtual reality in the treatment of eating and weight disorders. Psychological Medicine 2017; 47:2567–2568.^3^	3 studies	“Three different RCTs have shown at 1-year follow-up that VR for eating and weight disorders has a higher efficacy than the gold standard in the field, i.e. cognitive–behavioral therapy (CBT).”
Narrative review (mental health therapy)	Mishkind MC, Norr AM, Katz AC, et al. Review of virtual reality treatment in psychiatry: evidence versus current diffusion and use. Current Psychiatry Reports 2017; 19:80.^19^	Not reported	“More research is needed before VRE may be considered standard of care in some areas; however, for patients with PTSD or anxiety, and especially patients not responding or not willing to participate in traditional therapy, the use of VRE may be considered as an option. The use of VR for other conditions such as chronic pain, rehabilitation, and addictions also shows clinical promise.”
Systematic review (mental health assessment)	van Bennekom MJ, de Koning PP, Denys D. Virtual reality objectifies the diagnosis of psychiatric disorders: a literature review. Frontiers in Psychiatry 2017; 8:163.^4^	39 studies	“Nearly all VR environments studied were able to simultaneously provoke and measure psychiatric symptoms. Furthermore, in 14 studies, significant correlations were found between VR measures and traditional diagnostic measures. Relatively small clinical sample sizes were used, impeding definite conclusions.”
Narrative review (anxiety disorders)	Lindner P, Miloff A, Hamilton W, et al. Creating state of the art, next-generation virtual reality exposure therapies for anxiety disorders using consumer hardware platforms: design considerations and future directions. Cognitive Behaviour Therapy 2017; 46:404–420.^5^	Not reported	“While having been researched for decades and proven efficacious for the treatment of anxiety disorders, the pending and ongoing release of consumer-targeted VR hardware platforms signals an opportune time to develop the next generation of VR exposure therapies for widespread dissemination as self-help applications and integration into regular health care settings.”
Systematic review (mental health)	Massetti T, Crocetta TB, Silva TDD, et al. Application and outcomes of therapy combining transcranial direct current stimulation and virtual reality: a systematic review. Disability & Rehabilitation: Assistive Technology 2017; 12:551–559.^6^	11 studies	“The use of tDCS combined with VR showed positive results in both healthy and impaired patients including pain management. Future studies with larger sample sizes and homogeneous participants are required to confirm the benefits of tDCS and VR.”
Systematic review (mental health)	Jerdan SW, Grindle M, van Woerden HC, Kamel Boulos MN. Head-Mounted Virtual Reality and Mental Health: Critical Review of Current Research. JMIR Serious Games 2018; 6:e14.	82 studies	“Our review demonstrated that VR is effective in provoking realistic reactions to feared stimuli, particularly for anxiety; moreover, it proved that the immersive nature of VR is an ideal fit for the management of pain. However, the lack of studies surrounding depression and stress highlight the literature gaps that still exist.”
Systematic review and meta-analysis (acrophobia)	Arroll B, Wallace HB, Mount V, et al. A systematic review and meta-analysis of treatments for acrophobia. Med J Aust 2017; 206:263–267.	16 studies	“A range of therapies are effective for acrophobia in the short term but not in the long term. Many of the comparative studies showed equivalence between therapies, but this finding may be due to a type II statistical error. The quality of reporting was poor in most studies.”
Narrative review (psychosis)	Rus-Calafell M, Garety P, Sason E, et al. Virtual reality in the assessment and treatment of psychosis: a systematic review of its utility, acceptability and effectiveness. Psychological Medicine 2017 Jul 24 [Epub ahead of print].^7^	50 studies	“Virtual reality is a promising method to be used in the assessment of neurocognitive deficits and the study of relevant clinical symptoms. Furthermore, preliminary findings suggest that it can be applied to the delivery of cognitive rehabilitation, social skills training interventions and virtual reality-assisted therapies for psychosis.”
Systematic reviews (phobias)	Botella C, Fernández-Álvarez J, Guillén V, et al. Recent progress in virtual reality exposure therapy for phobias: a systematic review. Current Psychiatry Reports 2017; 19:42.^8^	11 studies	“VRET applications have become an effective alternative that can equal the results of traditional treatments for phobias from an efficacy point of view. However, they are also tools capable of enhancing the psychological treatment field.”
Narrative review (anxiety disorders)	Maples-Keller JL, Yasinski C, Manjin N, et al. Virtual reality-enhanced extinction of phobias and post-traumatic stress. Neurotherapeutics 2017; 14:554–563.^10^	Not reported	“VRE is consistent with models of extinction learning and provides several advantages for use within exposure-based interventions. Broadly, extant research provides support for the effectiveness of VRE in reducing symptoms of specific phobias and PTSD, with outcomes generally superior to waitlist controls and comparable with traditional exposure therapy.”
Meta-analysis (flight anxiety)	Cardoş RAI, David OA, David, DO. Virtual reality exposure therapy in flight anxiety: a quantitative meta-analysis. Computers in Human Behavior 2017; 72:371–380.^11^	11 studies	“Results pointed out significant overall efficiency of VRET in flight anxiety at post-test and follow-up. Analysis highlighted the superiority of VRET vs. control conditions at post-test and follow-up and the superiority of VRET vs. classical evidence-based interventions at post-test and follow-up.”
Narrative review (weight disorders)	Castelnuovo G, Pietrabissa G, Manzoni GM, et al. Cognitive behavioral therapy to aid weight loss in obese patients: current perspectives. Psychology Research & Behavior Management 2017; 10:165–173.^12^	Not reported	“Another current and future scenario where CBT could be improved in the management of obesity is represented by virtual reality (VR) applications, such as the VR-enhanced CBT that is a sort of enhanced CBT of obesity with a VR module focused on unlocking the negative memory of the body, changing its dysfunctional behavioral correlates, and managing negative emotional states.”
Narrative review (weight disorders)	Paul L, Van Der Heiden C, Hoek HW. Cognitive behavioral therapy and predictors of weight loss in bariatric surgery patients. Current Opinion in Psychiatry 2017; 30:474–479.^13^	Not reported	“Although empirical evidence is still scare, results show that CBT is effective in reducing disordered eating disorders and depression in bariatric patients. New techniques for applying CBR by virtual reality potentially make CBT more accessible and less costly.”
Systematic review (clinical medicine)	Dascal J, Reid M, Ishak WW, et al. Virtual reality and medical inpatients: a systematic review of randomized, controlled trials. Innovations in Clinical Neuroscience 2017; 14:14–21.^14^	11 studies	“Data from 11 eligible studies provide insight into three current medical applications of VR technology: pain distraction, eating disorders, and cognitive/motor rehabilitation. Overall, a majority of studies from the past decade found VR to be efficacious, easy to use, safe, and contributing to high patient satisfaction.”
Systematic review and meta-analysis (procedural pain)	Chan E, Foster S, Sambell R, Leong P. Clinical efficacy of virtual reality for acute procedural pain management: A systematic review and meta-analysis. PLoS ONE 2018; 13:e0200987.	20 studies	“VR may have a role in acutely painful procedures, however included studies were clinically and statistically heterogenous. Further research is required to validate findings, establish cost efficacy and optimal clinical settings for usage. Future trials should report in accordance with established guidelines.”
Narrative review (clinical medicine)	Li L, Yu F, Shi D, et al. Application of virtual reality technology in clinical medicine. American Journal of Translational Research 2017; 9:3867–3880.^15^	Not reported	“VR has shown to be effective in reduction of burn-induced pain and management of pain in other situations … Virtual reality exposure therapy and virtual reality cognitive behavior therapy have become effective choices for patients with anxiety disorders and other phobias like fear of flying, claustrophobia, acrophobia or generalized social phobia”
Narrative review (mental health)	Maples-Keller JL, Bunnell BE, Kim SJ, et al. The use of virtual reality technology in the treatment of anxiety and other psychiatric disorders. Harvard Review of Psychiatry 2017; 25:103–113.^9^	Not reported	“VR has emerged as a viable tool to help in a number of different disorders, with the most strength of evidence for use in exposure therapy for patients with anxiety disorders, cue exposure therapy for patients with substance use disorders, and distraction for patients with acute pain requiring painful procedures.”
Systematic review (eating disorders)	de Carvalho M, Dias T, Duchesne M, et al. Virtual reality as a promising strategy in the assessment and treatment of bulimia nervosa and binge eating disorder: a systematic review. Behavioral Sciences 2017; 7:43.^17^	19 studies	“Two different randomized, controlled trials have shown at one-year follow-up that VR had a higher efficacy than the gold standard in the field, i.e., cognitive behavioral therapy (CBT). In conclusion, based on the current available data VR-based environments may be considered a promising strategy for the assessment and treatment of BN and BED.”
Systematic review (clinical medicine)	Pourmand A, Davis S, Lee D, et al. Emerging utility of virtual reality as a multidisciplinary tool in clinical medicine. Games for Health Journal 2017; 6:263–270.^18^	45 studies	“These articles provide data, which strongly support the hypothesis that VR simulations can enhance pain management (by reducing patient perception of pain and anxiety), can augment clinical training curricula and physical rehabilitation protocols (through immersive audiovisual environments), and can improve clinical assessment of cognitive function (through improved ecological validity).”
Systematic review (autism)	Duffield TC, Parsons TD, Landry A, et al. Virtual environments as an assessment modality with pediatric ASD populations: a brief report. Child Neuropsychology 2017 Sep 13 [Epub ahead of print].^20^	5 studies	“Psychometric comparisons of these tools for the neuropsychological assessment of pediatric individuals with ASD are lacking as the current review demonstrated, although the use of VEs. This is a particularly important area of future research considering most identification, and thus testing, treatment, and training occur in childhood for ASD.”
Narrative review (pediatrics)	Parsons TD, Riva G, Parsons S, et al. Virtual reality in pediatric psychology. Pediatrics 2017; 140:S86–S91.^21^	Not reported	“VR can offer safe, repeatable, and diversifiable interventions that can benefit assessments and learning in both typically developing children and children with disabilities. Research has also pointed to VR's capacity to reduce children's experience of aversive stimuli and reduce anxiety levels.”
Systematic review (autism)	Mesa-Gresa P, Gil-Gomez H, Lozano-Quilis JA, Gil-Gomez JA. Effectiveness of virtual reality for children and adolescents with autism spectrum disorder: an evidence-based systematic review. Sensors (Basel) 2018; 18:pii:E2486.	31 studies	There is moderate evidence that VR-based treatments can help children with ASD. The lack of definitive findings does not allow us to state that VR-based treatments can improve the results of traditional treatments. Nevertheless, the promising results and the advantages of VR (especially considering ASD symptomatology) should encourage the scientific community to develop new VR-based treatments.
Systematic review (eating disorders)	Clus D, Larsen ME, Lemey C, Berrouiguet S. The use of virtual reality in patients with eating disorders: systematic review. J Med Internet Res 2018; 20:e157.	26 studies	Overall, VR techniques enable the evaluation of pathological eating behaviors and body image distortions. In addition to CBT, use of VR techniques by patients with eating disorders decreased their negative emotional responses to virtual food stimuli or exposure to their body shape.

All of the articles suggest that VR is suitable for the treatment of mental health problems and could make an important contribution in many different areas, from anxiety and eating disorders to psychosis and addiction.

The most common use of VR in behavioral health is for exposure therapy (VR exposure [VRE]). VRE is similar to classic exposure therapy^10,16,26^—the patient is exposed to a graded exposure hierarchy—with the only difference being that VR is substituted for other exposure techniques (e.g., in vivo or imaginal exposure). In the treatment of complex anxiety disorders, the use of VRE is often combined with other techniques such as breathing or relaxation exercises,^27^ attentional and autonomic control training,^28^ biofeedback,^29,30^ and/or cognitive restructuring.^31^

Five articles,^5,8,10^ including a meta-analysis,^11,16^ specifically explored the use of VRE in the treatment of anxiety disorders. The available data show that VR is able to reduce anxiety symptoms significantly in different anxiety disorders: phobias,^32^ post-traumatic stress disorders,^33^ panic disorder and agoraphobia,^34^ social anxiety disorders,^35^ psychological stress,^36^ and generalized anxiety disorders.^37^ The clinical outcome is generally superior to waitlist control conditions and comparable to in vivo exposure-based interventions.

A second group of five articles^3,12,13,17,23^ evaluated the efficacy of VR in the treatment of eating and weight disorders. In this field, VR is used in two different ways.^38^ First, VR cue exposure to critical stimuli (e.g., food or human bodies) allows both a reduction in the level of anxiety elicited by them and disruption of the reconsolidation of negative memories.^39,40^ Second, VR is used to facilitate the update of existing body representations.^41,42^ According to a recent theory,^43–47^ eating and weight disorders may be the outcome of a broader impairment in multisensory body integration that locks the individuals to an old memory of the body.^48^ In this view, even if the subject is able to lose weight after a diet, the multisensory impairment does not allow her/him to experience the new body and reduce the level of body dissatisfaction. VR allows a wrong representation of the body to be updated through two different strategies. In the first—“reference frame shifting”^49,50^—the subject re-experiences in VR a negative situation related to the body (e.g., teasing) in both the first and third person (e.g., seeing and supporting her/his avatar in the VR world). In the second—“body swapping”^51,52^—VR is used to induce the illusory feeling of ownership of a virtual body with a different shape and/or size. Even if the number of available controlled studies is less than for anxiety disorders, the field has rapidly evolved.^17^ Specifically, four different randomized controlled trials—one with eating disorders,^53^ one with morbid obesity,^54^ one with binge-eating,^55^ and one with binge-eating and bulimia^56^—have shown after 6-month and 12-month follow-ups that VR had a higher efficacy than the gold standard in the field, that is, cognitive–behavioral therapy.

A third group of three articles^20,21,24^ analyzed the use of VR in pediatric psychology, with a specific focus on VR applications for the assessment of children suspected of having autism spectrum disorder^57^ or other neurodevelopmental disorders^58,59^ (e.g., attention-deficit hyperactivity disorder). In this field, different from the previous ones, the level of clinical evidence available is still low, even if the existing data suggest moderate evidence about the effectiveness of VR-based treatments.^24^ In relation to this topic, another article specifically explored the use of VR for the assessment of psychiatric disorders,^4^ finding that virtual worlds are able to induce and assess psychiatric symptoms simultaneously, with significant correlations between VR measures and traditional diagnostic tools. Moreover, VR is also effective in assessing cue reactivity^60^: its use is able to increase subjective craving in smokers,^61,62^ alcohol drinkers,^63^ eaters,^64^ and cocaine-dependent individuals.^65^

Three final articles explored the use of VR in the assessment and treatment of psychosis^7^ and in pain management.^6,25^ For psychosis, the available studies confirm the efficacy of VR for the multimodal assessment of cognitive functioning,^7^ including social cognition/competence^66^ and hallucinations/paranoid ideations.^67^ For treatment, even if the available studies are very promising,^68–70^ there is a lack of randomized controlled trials demonstrating whether VR is more efficacious or efficient than other interventions.^7^

In relation to the use of VR for pain management, older systematic reviews^71,72^ demonstrated the efficacy of VR distraction^73–75^ for reducing experimental pain,^76^ as well as the one generated by burn injury care,^77–79^ chronic pain,^80–82^ and procedural pain.^83–85^ Hence, the first new one^6^ focused its analysis on the integrated use of VR with brain stimulation (transcranial direct-current stimulation) in pain management. Again, even if the level of clinical evidence is still low, a study^86^ demonstrated the efficacy of this approach in reducing the severity of neuropathic pain and various neuropathic pain subtypes. Finally, the second new one,^25^ suggests that VR may have a role in acutely painful procedures, even if further research is required.

Overall, this meta-review indicated that VR is a powerful clinical tool for behavioral health, able to provide effective assessment and treatment options for a variety of mental health disorders. Specifically, the 25 meta-analyses and systematic and narrative reviews indicated that VR compares favorably to existing treatments in anxiety disorders, eating and weight disorders, and pain management, with long-term effects that generalize to the real world. Moreover, they show the potential of VR as assessment tool with practical applications that range from social and cognitive deficits to addiction. Finally, they suggest a clinical potential in the treatment of psychosis and in the pediatric field, even if there is no definitive evidence for or against the use of VR.

## The Effectiveness of VR as a Clinical Tool

An open issue not directly addressed by most of these articles is why VR is an effective clinical tool. In many articles, attention is focused on the high level of control and customization allowed by this technology.^1,2,9,10,87^ VR allows the level of fit between the content of the exposure and the feared stimuli to be optimized. Moreover, using it, the therapist has a total control—limited only by the specific features of the used software—on the contents of the experience. Finally, it offers a safer and more private context for the patient that facilitates his/her engagement.

Another important point suggested by different articles is the level of “presence” provided by the virtual experience. In fact, VR provides a digital place to the individual where he/she can be placed and live a synthetic but realistic experience.^88^ As noted by some colleagues, VR can be considered an advanced imaginal system^89,90^: an advanced form of imagery that is as effective as reality in inducing experiences and emotions. For example, as demonstrated by a recent meta-analysis, presence and anxiety are associated with each other during VRE therapy for the treatment of anxiety.^91^ This allows a level of self-reflectiveness that is both more predictable and controllable than the one offered by reality, but higher than the one provided by memory and imagination.^1^ However, presence alone is necessary but not sufficient to achieve benefit from VR therapy.^92^ As noted by Price and Anderson, “The results support presence as a conduit that enabled phobic anxiety to be expressed during exposure to a virtual environment. However, presence was not supported as contributing to treatment outcome. This suggests feeling present during exposure may be necessary but not sufficient to achieve benefit from VR exposure.”^92(p750)^

A new argument that is introduced and discussed in this article is that VR shares with the brain the same basic mechanism: embodied simulations.^43,93^

## VR as Simulative Technology

An increasingly popular hypothesis—predictive coding^94–96^—suggests that the brain actively maintains an internal model (simulation) of the body and the space around it, which provides predictions about the expected sensory input and tries to minimize the amount of prediction errors (or “surprise”). An in-depth discussion of these concepts is not offered here because authoritative and thorough accounts have been provided elsewhere.^94–99^ However, herein, the focus is on the concept of simulation introduced by this paradigm to understand better the links between the brain and VR.

One of the main tenets of predictive coding is that to regulate and control the body in the world effectively, the brain creates an embodied simulation of the body in the world. There are two main characteristics of this simulation. First, different from other internal models used in cognitive science—such as Tolman's cognitive maps or Johhson–Laird's internal models—they are simulations of sensory motor experiences. In this view, they include visceral/autonomic (interoceptive), motor (proprioceptive), and sensory (e.g., visual, auditory) information. Second, embodied simulations reactivate multimodal neural networks, which have produced the simulated/expected effect before.

This approach is used not only for actions, but also for concepts and emotions. Specifically, a concept is a group of distributed multimodal “patterns” of activity across different populations of neurons (motor, somatosensory, limbic, and frontal areas) that support a goal achievement.^100,101^ So, the simulation of a concept involves its reenactment in modality-specific brain areas. Moreover, the brain uses emotion concepts to categorize sensations. As underlined by Barrett, “That is, the brain constructs meaning by correctly anticipating (predicting and adjusting to) incoming sensations. Sensations are categorized so that they are (a) actionable in a situated way and therefore (b) meaningful, based on past experience. When past experiences of emotion (e.g., happiness) are used to categorize the predicted sensory array and guide action, then one experiences or perceives that emotion (happiness).”^100(p9)^ In this view, the feeling of presence in a space can be considered as an evolutive tool used to track the difference between the predicted sensations and those that are incoming from the sensory world, both externally and internally.^93,102,103^

VR works in a similar way: it uses computer technology to create a simulated world that individuals can manipulate and explore as if they were in it. In other words, the VR experience tries to predict the sensory consequences of your movements, showing to you the same scene you will see in the real world. Specifically, VR hardware tracks the motion of the user, while VR software adjusts the images on the user's display to reflect the changes produced by the motion in the virtual world. To achieve it, like the brain, the VR system maintains a model (simulation) of the body and the space around it. This prediction is then used to provide the expected sensory input using the VR hardware. Obviously, to be realistic, the VR model tries to mimic the brain model as much as possible: the more the VR model is similar to the brain model, the more the individual feels present in the VR world.^93,104^

## VR as Embodied Technology

As has just been seen, the brain creates multiple multisensory simulations to predict^100^: (a) upcoming sensory events both inside and outside the body, and (b) the best action to deal with the impending sensory events. Moseley et al. suggested that these simulations are integrated with sensory data in the “body matrix,” a coarse supramodal multisensory representation of the body and the space around it.^105–107^ Specifically, the contents of the body matrix are defined by top-down predictive signals, integrating the multisensory (motor and visceromotor) simulations of the causes of perceived sensory events.^108^ The different simulations are then ranked and included in the body matrix according to their relevance for the intentions of the self (selective attention). At the same time, the content and the priority of the different simulations are corrected by bottom-up prediction errors that signal mismatches between predicted and actual contents of sensory events.^109^

At the end of this process, the body matrix defines where the self is present, that is, in the body that our brain considers as the most likely to be its one.^110–112^ As underlined by Apps and Tsakiris, “The mental representation of the physical properties of one's self are, therefore, also probabilistic. That is, one's own body is the one that has the highest probability of being ‘me,’ since other objects are probabilistically less likely to evoke the same sensory inputs. In short, the notion that there is a ‘self’ is the most parsimonious and accurate explanation for sensory inputs.”^110(p88)^

If presence in the body is the outcome of different embodied simulations, and VR is a simulation technology, this suggests the possibility of altering the experience of the body by designing targeted virtual environments.^113^ In this view, VR can be defined as an “embodied technology” for its possibility of modifying the embodiment experience of its users.^114–116^ As noted by Riva et al., “using VR, subjects can experience the synthetic environment as if it was ‘their surrounding world’ (*incarnation*: the physical body is within a virtual environment) or can experience their synthetic avatars as if they were ‘their own body’ (*embodiment*: the physical body is replaced by the virtual one).”^1(p9)^ In other words, VR is able to fool the predictive coding mechanisms used by the brain generating the feeling of presence in a virtual body and in the digital space around it.

Up to now, VR has been used to simulate external reality, that is, to make people feel “real” what is actually not really there (i.e., the environment). However, the ability of VR to fool the predictive coding mechanisms that regulate the experience of the body also allows it to make people feel “real” what they are not. In other words, VR can offer new ways for structuring, augmenting, and/or replacing the experience of the body for clinical goals.^114–116^ Moreover, it may offer new embodied ways for assessing the functioning of the brain^117,118^ by directly targeting the processes behind real-world behaviors.^119–121^

But what is the real clinical potential of VR as an embodied technology? According to neuroscience, the body matrix^105,106,122,123^ serves to maintain the integrity of the body at both the homeostatic and psychological levels by supervising the cognitive and physiological resources necessary to protect the body and the space around it. Specifically, the body matrix plays a critical role in high-end cognitive processes such as motivation, emotion, social cognition, and self-awareness,^124–126^ while exerting a top-down modulation over basic physiological mechanisms such as thermoregulatory control^127,128^ and the immune system.^123^

In this view, different authors^114,116,129,130^ have recently suggested that an altered functioning of the body matrix and/or its related processes might be the cause of different neurological and psychiatric conditions. If this is true, VR can be the core of a new trans-disciplinary research field—embodied medicine^115,116^—the main goal of which is the use of advanced technology for altering the body matrix, with the goal of improving people's health and well-being.

As has been seen in the first section of this article, two different VR embodiment techniques—body swapping^51,52^ and reference frame shifting^49,50^—are currently used in the treatment of eating and weight disorders. The first one, body swapping, replaces the contents of the bodily self-consciousness with synthetic ones (synthetic embodiment). This has been used in eating and weight disorders to improve the experience of the body in both clinical (anorexia and morbid obesity)^131,132^ and non-clinical subjects.^133–135^ Nevertheless, the potential of this approach is wider.^136^ For example, it may offer a non-pharmacological way to reduce chronic pain. As has been seen in the first section of this article, VR distraction is effectively used to reduce acute pain. Nevertheless, according to Tsay et al., “available findings present compelling evidence for a novel multisensory and multimodal approach to therapies for chronic pain disorders”^137(p249)^ In this view, the use of VR embodiment may offer new treatment options for pain management.^138–140^ Some studies have suggested the possibility of using VR body swapping to improve body perception disturbance in patients with complex regional pain syndrome.^141,142^

The second technique, reference frame shifting, structures the individual's bodily self-consciousness through the focus and reorganization of its contents (mindful embodiment).^50,143^ It has been successfully used in different randomized trials in patients with eating and weight disorders^54,55^ to update the contents of their body memory. But again, its applications are probably wider. For example, Osimo et al. integrated body swapping (in the avatar of Sigmund Freud) and reference frame shifting to improve mood and happiness in a non-clinical sample.^143^

A final emerging approach is the use of VR to augment the bodily experience through the awareness of internal (and difficult to sense) bodily information, or the mapping of a sensory channel to a different one—for example vision to touch or to hearing (augmented embodiment).^144,145^ For example, Suzuki et al.^146^ implemented an innovative “cardiac rubber hand illusion” that combined computer-generated augmented reality with feedback of interoceptive information. Their results showed that the virtual-hand ownership is enhanced by cardio-visual feedback in time with the actual heartbeat, supporting the use of this technique to improve emotion regulation.

## VR as Cognitive Technology

VR is an embodied technology for its ability to modify the experience of the body. However, the body is not simply an object like any other; it has a special status.^93,147,148^ It is perceived in a multisensory way, from the outside (exteroception, the body perceived through the senses) as well as from within (inner body, including interoception, the sense of the physiological condition of the body; proprioception, the sense of the position of the body/body segments; and vestibular input, the sense of motion of the body) and from memory. This is true also for the simulative code used by the brain for creating concepts. As has been seen before, it integrates visceral/autonomic (interoceptive), motor (proprioceptive), and sensory information. If concepts are embodied simulations, and VR is an embodied technology, it should be possible to facilitate cognitive modeling and change by designing targeted virtual environments able to modify concepts both from outside and from inside.^114^

Nevertheless, there is a critical shortcoming that at the moment is limiting this possibility: VR simulates the external world/body but not the internal one. In fact, actual VR technology is very effective in reproducing the exteroceptive (external) features of the body using vision and hearing, but less effective in reproducing the other senses (i.e., touch and smell^149^). It is partially effective in reproducing the proprioceptive (motor) features of the body using haptic technologies,^150^ but it is not yet able to reproduce the interoceptive/vestibular (internal) features of the body.

**FIG. 2.**
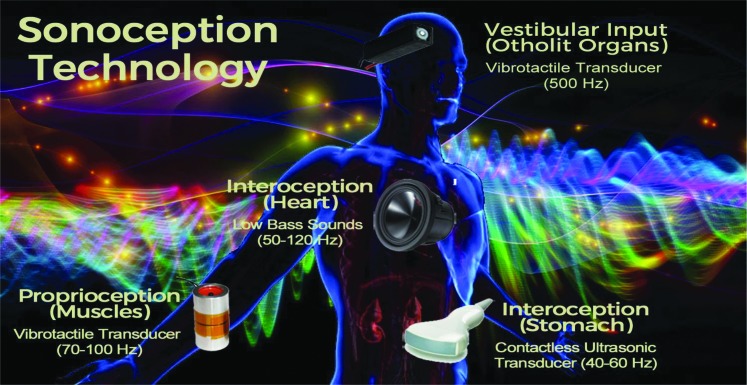
The technology of “sonoception.”

Recently, Riva et al.^116^ introduced the concept of “sonoception,” a novel noninvasive technological paradigm based on wearable acoustic and vibrotactile transducers, as a possible approach to structure, augment, and/or replace the contents of the inner body. This approach should be able to modulate the inner body (interoception, proprioception, and vestibular input) through the stimulation of both mechanoreceptors in different parts of the body—the stomach, the heart, the muscles—and the otolith organs of the vestibular system (see Fig. 2).

The first outcome of an integrated VR platform able to simulate both the external and the inner world is the possibility of structuring, augmenting, and/or replacing all the different experiential aspects of bodily self-consciousness, with clinical applications in the treatment of psychiatric disorders, such as depression^151,152^ or schizophrenia,^153–155^ and neurological disorders, such as chronic pain^137,156^ and neglect.^157,158^

The final long-term outcome of this possibility may be the embodied virtual training machine described by the science-fiction thriller The Matrix. In this movie, the heroes, Trinity and Neo, learned how to fight martial-arts battles and drive motorcycles and helicopters by experiencing the bodily processes and concepts related to the skill through an embodied simulation.

## Conclusions

The first article discussing a VR application in the field of behavioral health was published in 1995.^159^ Now, more than 20 years later, VR is a reality in this field. This is the result of a meta-review presented in this article assessing the meta-analyses and systematic and narrative reviews published in this field in the last 22 months. Twenty-five different articles have demonstrated the clinical potential of this technology in both the diagnosis and the treatment of mental health disorders. Specifically, they indicate that VR compares favorably to existing treatments in anxiety disorders, eating and weight disorders, and pain management, with long-term effects that generalize to the real world.

But why is VR so effective? Here, the following answer is suggested: VR shares with the brain the same basic mechanism—embodied simulations.

According to neuroscience, to regulate and control the body in the world effectively, the brain creates an embodied simulation of the body in the world used to represent and predict actions, concepts, and emotions. Specifically, it is used to predict: (a) upcoming sensory events both inside and outside the body, and (b) the best action to deal with the impending sensory events.^100^ There are two main characteristics of this simulation. First, it simulates sensory motor experiences, including visceral/autonomic (interoceptive), motor (proprioceptive), and sensory (e.g., visual, auditory) information. Second, embodied simulations reactivate multimodal neural networks which have produced the simulated/expected effect before.

VR works in a similar way: the VR experience tries to predict the sensory consequences of the individual's movements, providing to him/her the same scene he/she will see in the real world. To achieve this, the VR system, like the brain, maintains a model (simulation) of the body and the space around it.

If presence in the body is the outcome of different embodied simulations, and VR is a simulation technology, this suggests the possibility of altering the experience of the body by designing targeted virtual environments.^113^ In this view, VR can be defined as an “embodied technology” for its possibility of modifying the embodiment experience of its users.^114–116^ In other words, VR is able to fool the predictive coding mechanisms used by the brain, generating the feeling of presence in a virtual body and in the digital space around it.

Moreover, if concepts are embodied simulations, and VR is an embodied technology, it should be possible to facilitate cognitive modeling and change by designing targeted virtual environments able to modify concepts from both outside and inside.^114^

Nevertheless, at the moment, there is a critical shortcoming that is limiting this possibility: VR simulates the external world/body but not the internal one. Recently, Riva et al.^116^ introduced the concept of “sonoception” (www.sonoception.com), a novel noninvasive technological paradigm based on wearable acoustic and vibrotactile transducers able to stimulate both mechanoreceptors in different parts of the body—the stomach, the heart, the muscles—and the otolith organs of the vestibular system (see Fig. 2). The first outcome of this approach is the development of an interoceptive stimulator that is both able to assess interoceptive time perception in clinical patients^160^ and to enhance heart rate variability (the short-term vagally mediated component—rMSSD) through the modulation of the subjects' parasympathetic system.^161^ The integration of these technologies with VR in a multisensory simulative platform will allow the modulation of both the external and internal bodily information, to structure, augment and/or replace the contents of our bodily self-consciousness.

In conclusion, even if VR is already a reality in behavioral health, the possibility of using it to simulate both the external and internal world may open new clinical options in the near future able to target the experience of the body and its related processes directly. Psychosomatics is an interdisciplinary field that explores the relationships between psychosocial, behavioral factors, and bodily processes. The long-term goal of the vision presented in this article is the use of simulative technologies—both simulating the external world and the internal one—to reverse engineer the psychosomatic processes that connect mind and body. If achieved, this perspective will provide a radically new meaning to the classical Juvenal's Latin dictum “Mens sana in corpore sano” (a healthy mind in a healthy body) by allowing a new trans-disciplinary research field—“Embodied Medicine”^115,116^—that will use advanced multisensory technologies to alter bodily processes for enhancing homeostasis and well-being.
